# Mono-ADP-ribosylation of H3R117 traps 5mC hydroxylase TET1 to impair demethylation of tumor suppressor gene *TFPI2*

**DOI:** 10.1038/s41388-018-0671-8

**Published:** 2019-01-16

**Authors:** Ming Li, Yi Tang, Qingshu Li, Ming Xiao, Yaying Yang, Yalan Wang

**Affiliations:** Department of Pathology, Molecular Medicine and Cancer Research Center, Chongqing Medical University, Chongqing, 400016 China

**Keywords:** DNA methylation, Colorectal cancer

## Abstract

Recently, nuclear poly-ADP-ribosylation had aroused research interest in epigenetics, but little attempt to explore functions of mono-ADP-ribosylation of histone, the major formation of histone ADP-ribosylated modification. We have previously reported a novel mono-ADP-ribosylation of H3R117, which promoted proliferation of LoVo cells. Here we showed that mono-ADP-ribosylated H3R117 of LoVo cells depressed demethylation of tumor suppressor *TFPI2* promoter by suppressing TET1 expression and adjusting H3K9me3 enrichment of *TFPI2* promoter to attenuate affinity of TET1, besides, since high H3K27me3 level was associated with hypermethylation, mono-ADP-ribosylated-H3R117-depended-H3K27me3 of *TFPI2* promoter may contribute to hypermethylation of *TFPI2*. However, H3R117A mutation increased poly-ADP-ribosylated modification of *TET1* promoter not *TFPI2* promoter, which resulted in boosting transcription and expression of TET1 by altering DNA methylated modification, chromatin accessibility, and histone-methylated modification of *TET1* promoter, while knockout TET1 of H3R117A LoVo cells directly led to hypermethylation of *TFPI2* promoter and depression of TFPI2 secretion as well as enhanced proliferation, suggested that TET1 played a key role in demethylation of *TFPI2*, production of TFPI2, and cell proliferation. Bioinformatics analyses reveal prevalent hypermethylation of *TFPI2* was an early event in tumorigenesis of colorectal caner, and expression of TET1 and TFPI2 was positive correlation in colorectal cancer and normal tissue. These data suggested that mono-ADP-ribosylation of H3R117 upregulated methylation of *TFPI2* by impact TET1, since hypermethyaltion of *TFPI2* was an early event in tumorigenesis, selectively target mono-ADP-ribosylation of H3R117 deficiency could be a feasible way to block tumorigenesis of colorectal cancer.

## Introduction

Tissue factor pathway inhibitor-2 (TFPI2), a Kunitz-type serine proteinase inhibitor, can inhibit a variety of serine proteases including factor VIIa/tissue factor, factor Xa, plasmin, trypsin, chymotryspin, and plasma kallikrein. During the past decade studies, *TFPI2* has been identified as a tumor suppressor gene (TSG) in several types of cancer, including colorectal cancer (CRC) [1–3]. *TFPI2* methylation frequently existed in CRC patients’ sera [4] and stool [5]. Moreover, hypermethylated *TFPI2* was associated with recurrence and early stage of CRC [6], besides, *TFPI2* was significant in CRC patients’ sera with large, poorly differentiated carcinoma, deep invasion, lymph node metastasis, or distant metastasis [4]. Additionally, Hibi et al. confirmed that detection of methylated *TFPI2* in serum DNA was derived from CRC [7]. Thus, *TFPI2* methylation was relevant to tumorigenesis and prognosis of CRC, but there are little strategies were provided to prevent *TFPI2* hypermethylation in CRC.

DNA methylation is an epigenetic marker, which is important for controlling gene expression. While ten-eleven translocation (TET) family mediates the sequential oxidation of 5-methylcytosine (5mC) to 5-hydroxymethylcytosine (5hmC), then further to 5-formylcytosine and 5-carboxylcytosine, leading to eventual DNA demethylation [8–12]. Among TET family, the most-studied member is TET1. In CRC and some CRC cell lines, mRNA expression of TET1 and global 5hmC level were detected lower than normal tissue or normal colon cells [13–17]; TET1 was capable to react with TSGs by depressing DNA methylation [14, 18], suggesting that enhancement of TET1 expression could be a feasible way of preventing methylation of TSGs of CRC.

ADP-ribosylation is an important post-translational modification of protein. It alters the functional proteins or recruits other proteins by providing a scaffold on the modified proteins and thus regulates several cellular processes. Recently, the function of nuclear ADP-ribosylation in epigenetics became a novel focus [19]. Besides, Ciccarone et al. reported that nuclear poly-ADP-ribosylation was a key positive epigenetic regulator of TET1 transcription by maintaining an active chromatin state of promoter [20]. Interestingly, it was not poly- but mono- or oligo-ADP-ribosylation that was the primary type of histone ADP-ribosylation, while histone poly-ADP-ribosylation was a responsible result to some stress condition [21]; however, the contribution of histone mono-ADP-ribosylation for transcription of TET1 is largely unknown as an epigenetic event.

Since a specific site of histone ADP-ribosylation could cause specific nucleosome structure changes, identification of the target amino acids of histone ADP-ribosylation will further illuminate the interaction between histone and DNA [22]. In previous research, we detected mono-ADP-ribosylation on H3R117 in LoVo cells by liquid chromatography-tandem mass spectrometry (LC-MS/MS), and mutated arginine (R) 117 of H3 to non-ADP-ribosylated alanine (A), as H3R117A LoVo cells, observing depressing proliferation of H3R117A LoVo cells by mutation of H3 mono-ADP-ribosylated R117 [23]. Therefore, we speculated that mono-ADP-ribosylated modification on H3R117 definitely did some contribution to alter chromatin microenvironment of some specific genes and induced subsequently influence malignant biological behavior of cancer cells. However, further research needs to be done.

Thus, in this study, we assessed the effect of mono-ADP-ribosylated H3R117 of LoVo cells on methylation of TSG *TFPI2*, which was associated with tumorigenesis and prognosis of CRC, and discussed potential epigenetic mechanism involving TET1.

## Results

### Mono-ADP-ribosylation of H3R117 impacted methylated and hydroxymethylated modification of TSG *TFPI2* as well as secretion of TFPI2

**Fig. 1:**
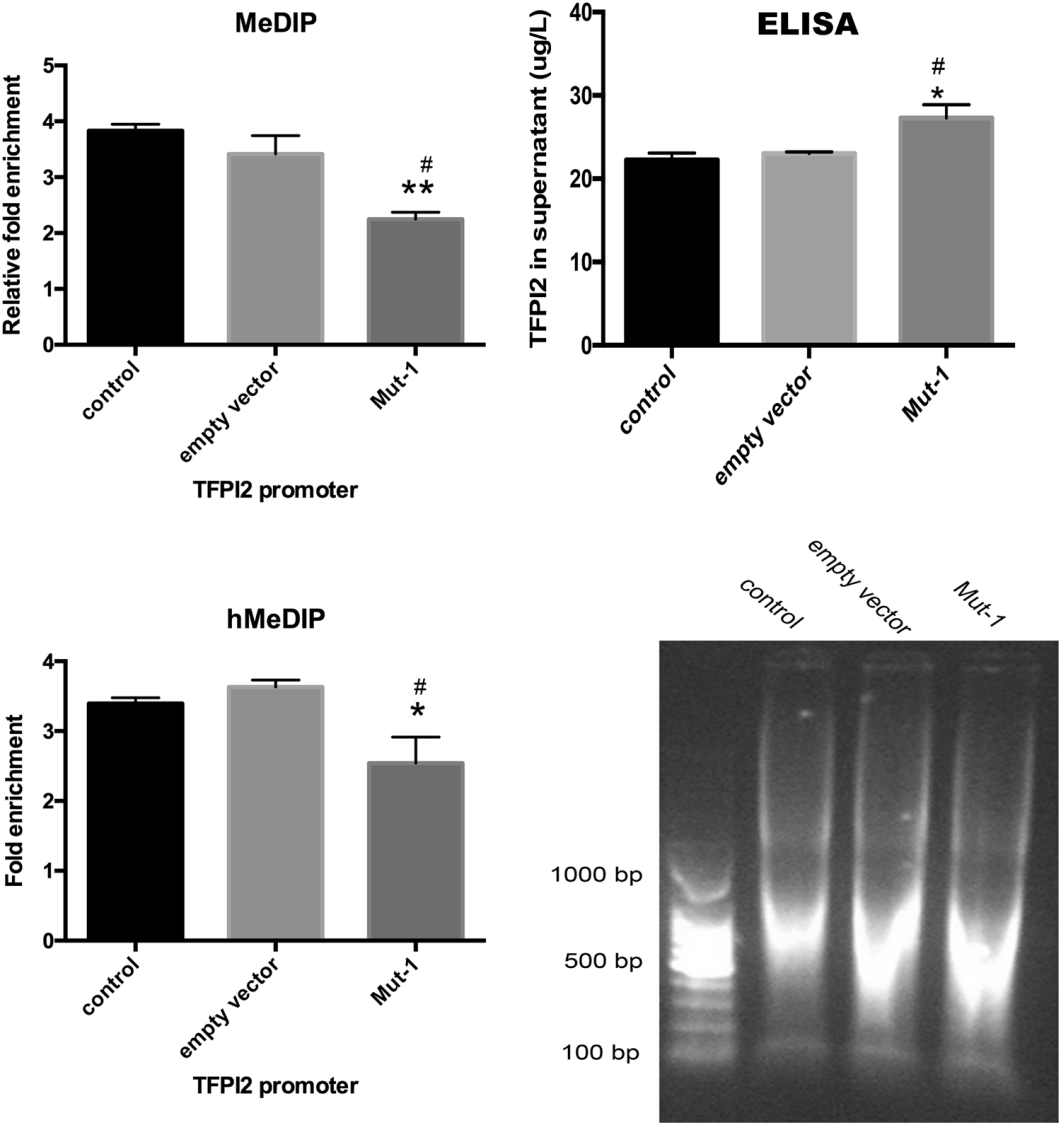
Mono-ADP-ribosylation of H3R117 altered methylated and hydroxymethylated modification of *TFPI2* promoter DNA as well as secretion of TFPI2. DNA was extracted from cultured cells and sheared into length of 200–1000 bp DNA by sonication. Then, sheared DNA was immunoprecipitated with anti-5-methylcytosine or anti-5-hydroxymethylcytosine. Sample was amplified with primer specific for *TFPI2* promoter. For Methylated DNA Immunoprecipitation-quantitative PCR (MeDIP-qPCR), relative fold enrichment was calculated as the ratio of amplification efficiency of the MeDIP sample over that of non-immune IgG as of the following the formula: relative fold enrichment = 2^(Ct input − Ct sample)^/2^(Ct input − Ct IgG)^. For Hydroxymethylated DNA Immunoprecipitation (hMeDIP)-qPCR, fold enrichment was calculated as the ratio of amplification efficiency of the hMeDIP sample over that of non-immune IgG, fold enrichment % = 2^(Ct IgG − Ct sample)^ × 100%. Since TFPI2 was a secretory protein, enzyme-linked immunosorbent assay (ELISA) kit was used to detect the secretion of TFPI2 in each group, and absorbance was measured by ELISA reader at optical density 450 nm (*P < 0.05 vs control, ^#^*P* < 0.05 vs empty vector, **P < 0.001 vs control)

DNA of each group was extracted by using Methylated DNA Immunoprecipitation (MeDIP) kit to quantify with size range of DNA (200–1000 base pairs) (Fig. 1). To evaluate effect of mono-ADP-ribosylation of H3R117 on methylated modification level of *TFPI2*, we used MeDIP-quantitative PCR (qPCR) to measure the enrichment of 5mC on TFPI2 promoter. Inspiringly, quantitation of these observations showed a lower methylation level in H3R117A mutant LoVo cells compared with untreated or empty vector-transfected LoVo cells, accordingly, higher secretion of TFPI2 was detected in H3R117A mutant LoVo cells with respect to other two control groups by enzyme-linked immunosorbent assay (ELISA) (Fig. 1), presenting mono-ADP-ribosylated H3R117 did the contribution to hypermethylated modification of *TFPI2* promoter and inhibited secretion of TFPI2 on LoVo cells. We further analyzed hydroxymethylated modification level of *TFPI2* promoter by applying hydroxymethylated DNA immunoprecipitation (hMeDIP)-qPCR; interestingly, we found hydroxymethylation of *TFPI2* promoter in H3R117A LoVo cells was reduced with respect to control or empty vector-transfected LoVo cells as well (Fig. 1), demonstrating that hydroxymethylation is an independent epigenetic modification of methylation.

### Mono-ADP-ribosylation of H3R117 inhibited affinity of TET1 to *TFPI2* promoter in LoVo cells by modulating H3K9me3 modification rather than influence of poly-ADP-ribosylation

**Fig. 2:**
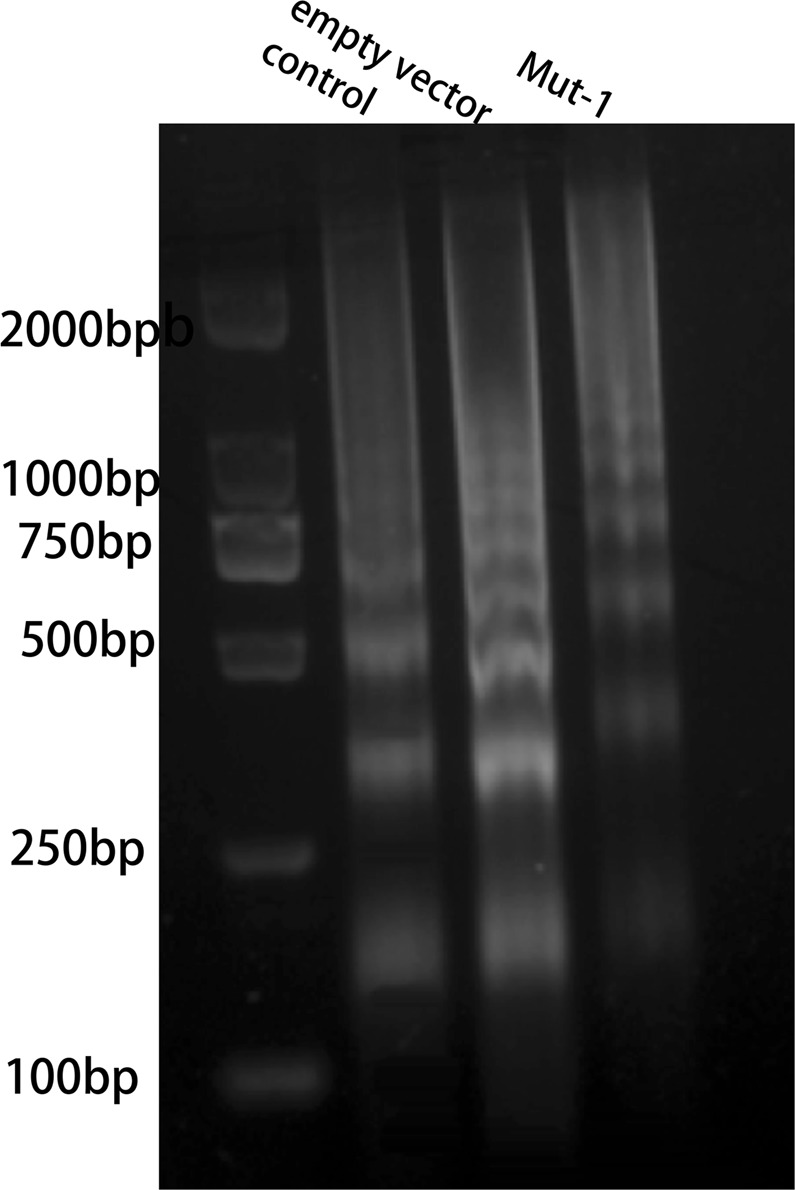
DNA shearing of chromatin immunoprecipitation (ChIP) assay. Sufficient DNA was extracted from each group according to ChIP kit, and micrococcal nuclease was added to digest DNA into 150–900 bp fragments

**Fig. 3:**
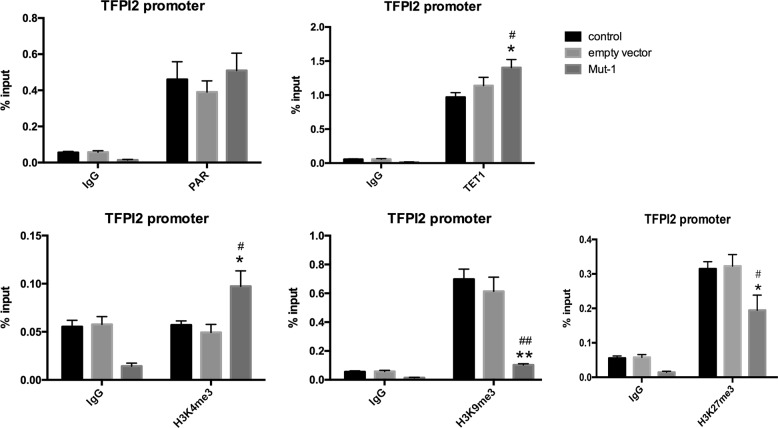
Chromatin immunoprecipitation (ChIP) for *TFPI2* promoter. This figure showed the enrichment of PAR/TET1/H3K4me3/H3K9me3/H3K27me3 on *TFPI2* promoter in each group by ChIP assay followed by quantitative PCR analysis. % input = 2% × 2^(Ct 2% input sample − Ct IP sample)^ (**P < 0.001 vs control, ^##^*P* < 0.001 vs empty vector, *P < 0.05 vs control, ^#^*P* < 0.05 vs empty vector)

Sufficient DNA of each group was extracted by using chromatin immunoprecipitation (ChIP) kit to quantify with size range of DNA (200–1000 base pairs) (Fig. 2). Consistent with lower methylation of *TFPI2*, ChIP assay results revealed that more enrichment of TET1 was observed on *TFPI2* promoter in H3R117A LoVo cells compared with control and empty vector-transfected LoVo cells, suggesting that TET1 enriched in *TFPI2* promoter and hydroxylated 5mC leading to a decrease of methylation of *TFPI2* promoter in H3R117A LoVo cells. Nevertheless, ChIP assay showed no significant difference of poly(ADP-ribose) (PAR) enrichment on the *TFPI2* promoter among H3R117A LoVo cells, untreated, and empty vector-transfected LoVo cells (Fig. 3), indicating that mono-ADP-ribosylation of H3R117 did not change poly-ADP-ribosylation of *TFPI2* promoter, or, in other words, it precluded the influence of poly-ADP-ribosylation on DNA methylation of *TFPI2* promoter.

Considering H3K9me3-marked genes were not targeted for hyper- or hypomethylation by TET proteins [24], we assessed enrichment of H3K9me3 on *TFPI2* promoter by ChIP-qPCR. The result showed barely enrichment of H3K9me3 on *TFPI2* promoter in H3R117A LoVo cells compared with control and empty vector-transfected LoVo cells (Fig. 3), and demonstrated that loss of H3K9me3 exposed target sites to TET proteins, allowing TET proteins to bind on *TFPI2* promoter and hydroxylate 5mC in H3R117A LoVo cells. That was mono-ADP-ribosylation of H3R117 protected DNA hypermethylation from TET1 with modification of H3K9me3 on *TFPI2* promoter.

### Mono-ADP-ribosylation of H3R117 impacted H3K4me3 and H3K27me3 modifications on *TFPI2* promoter in LoVo cells

Zhou et al. reported that loss of TET1 increased histone H3K27me3 causing repression of the target gene E-cadherin in DLD1 colon cancer cells [25]. Accordingly, our data showed that relative lower affinity of TET1 presented higher enrichment of H3K27me3 on *TFPI2* promoter in control LoVo cells and empty vector LoVo cells, comparing with H3R117A mutation of LoVo cells (Fig. 3), suggesting that mono-ADP-ribosylation of H3R117 impacted H3K27me3 enrichment on *TFPI2* promoter in LoVo cells by inhibiting affinity of TET1.

Putiri et al. proposed that even occupied by TET1, lack of 5hmC appeared commonly at promoters enriched with H3K4me3, due to TET2 played a major role in 5hmC removal on these loci [24]. Thus, to explain reduced hydroxymethylation of *TFPI2* promoter on H3R117A mutant LoVo cells occupying with more TET1 enzyme, we detected H3K4me3 modification of *TFPI2* promoter. Our data showed a significant increase in the H3K4me3 level of the *TFPI2* promoter on H3R117A mutant LoVo cells with respect to mono-ADP-ribosylated H3R117 LoVo cells (Fig. 3), suggesting that, although there is high enrichment of TET1 at the *TFPI2* promoter, lower hypohydroxymethylation of *TFPI2* promoter was shown in H3R117A mutant LoVo cells, probably owing to the relatively higher enrichment of H3K4me3 on the promoter inducing TET2 to remove 5hmC from the *TFPI2* promoter.

### Mono-ADP-ribosylation of H3R117 inhibited transcription and expression of TET1

**Fig. 4:**
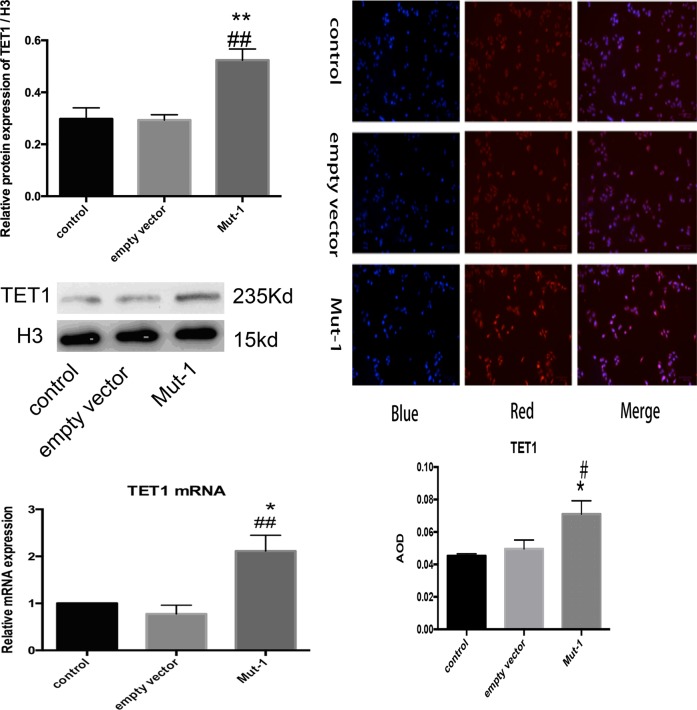
Mono-ADP-ribosylation of H3R117 decreased mRNA and protein expression of TET1. Quantitative PCR was applied for detecting mRNA level of TET1 for each group, while western blot and immunofluorescence were applied for measuring protein expression of TET1 for each group. The grayscale values of the bands of mutant group TET1/H3 were compared with those of the control band TET1/H3 and the ratios were subjected to statistical analysis. For immunofluorescence, average optical density was measured by ImageJ software (*P < 0.05 vs control, **P < 0.001 vs control, ^##^*P* < 0.001 vs empty vector)

To evaluate the mRNA and protein levels of TET1, we performed Quantitative real-time PCR (RT-qPCR), western blot, and immunofluorescence. Our results showed that, compared with untreated or empty vector-transfected LoVo cells, H3R117A mutant LoVo cells were detected with significantly higher mRNA levels of TET1 (Fig. 4). Accordingly, western blot analysis demonstrated greater TET1 protein level in H3R117A LoVo cells than other two control groups of LoVo cells (Fig. 4), meanwhile, immunofluorescence showed higher average optical density (OD) in the nucleus of H3R117A mutant LoVo cells comparing with control and empty vector group (Fig. 4), suggesting that mono-ADP-ribosylation of H3R117 inhibited expression of TET1 in LoVo cells.

### Mono-ADP-ribosylation of H3R117 influenced DNA methylation but not hydroxymethylation of *TET1* promoter

**Fig. 5:**
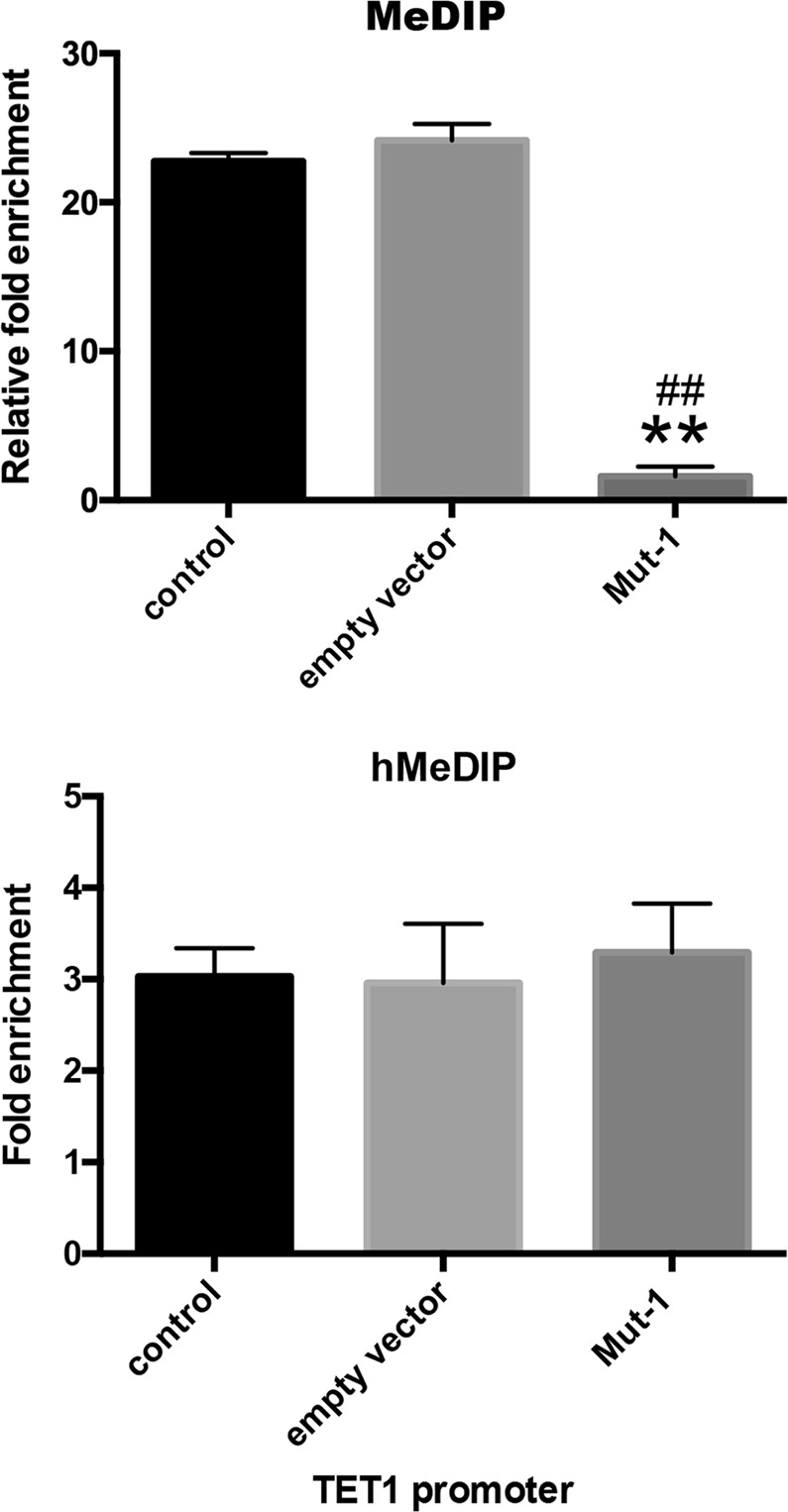
Mono-ADP-ribosylation of H3R117 altered methylated and hydroxymethylated modification of *TET1* promoter. Methylated DNA Immunoprecipitation (MeDIP) kit-extracted DNA was immunoprecipitated with anti-5mC or anti-5hmC. Sample was amplified with primer specific for *TET1* promoter. For MeDIP-quantitative PCR, relative fold enrichment was calculated as the ratio of amplification efficiency of the MeDIP sample over that of non-immune IgG as of the following the formula: relative fold enrichment = 2^(Ct input − Ct sample)^/2^(Ct input − Ct IgG)^. For Hydroxymethylated DNA Immunoprecipitation (hMeDIP)-qPCR, fold enrichment was calculated as the ratio of amplification efficiency of the hMeDIP sample over that of non-immune IgG, fold enrichment % = 2^(Ct IgG − Ct sample)^ × 100% (** *P* < 0.001 vs control, ^##^*P* < 0.001 vs empty vector)

We measured the alters of methylated and hydroxymethylated level of *TET1* promoter by MeDIP-qPCR and hMeDIP-qPCR. Consistent with boosted mRNA and protein levels of TET1, 5mC enrichment of *TET1* promoter was reduced while 5hmC enrichment of *TET1* promoter was not changed in H3R117A LoVo cells with respect to untreated or empty vector-transfected LoVo cells (Fig. 5), illustrating mono-ADP-ribosylation of H3R117 facilitated hypermethylation but not hydroxymethylation of *TET1* promoter.

### Mono-ADP-ribosylation of H3R117 influenced chromatin microenvironment of *TET1* promoter

**Fig. 6:**
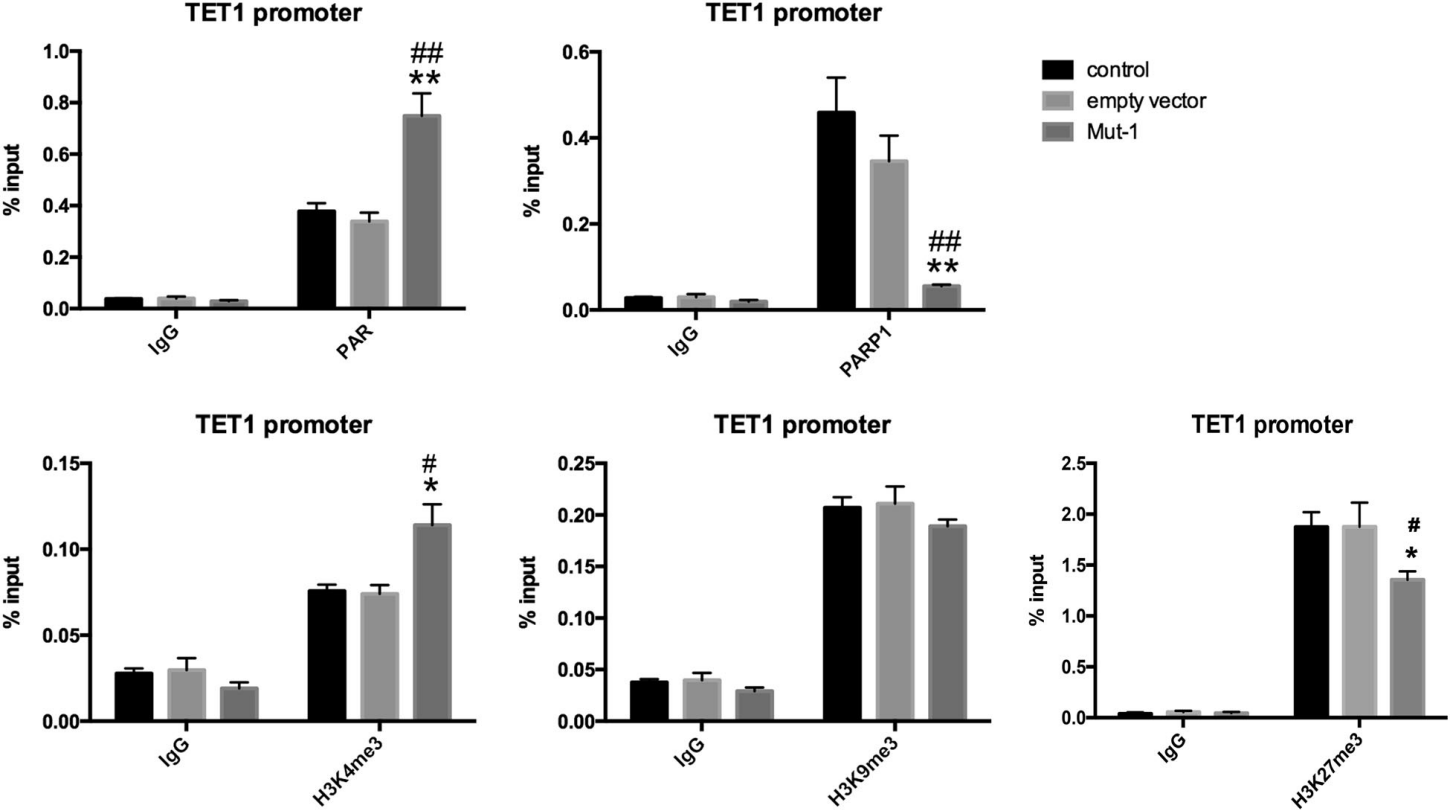
Chromatin immunoprecipitation (ChIP) for *TET1* promoter. This figure showed the enrichment of PAR/PARP1/H3K4me3/H3K9me3/H3K27me3 on *TET1* promoter in each group by ChIP assay followed by quantitative PCR analysis. % input = 2% × 2^(Ct 2% input sample − Ct IP sample)^ (mean %input) ± SD (*n* = 3) (**P < 0.001 vs control, ^##^*P* < 0.001 vs empty vector, *P < 0.05 vs control, ^#^*P* < 0.05 vs empty vector)

ChIP assay was used to detect the chromatin environment of *TET1* promoter. We found that PAR was abundant on *TET1* promoter in H3R117A LoVo cells with respected to untreated and empty vector-transfected LoVo cells, suggesting that mono-ADP-ribosylated H3R117 mutation increased poly-ADP-ribosylated modification on *TET1* promoter. Meanwhile, increased enrichment of H3K4me3 and decreased enrichment of H3K27me3 were discovered on *TET1* promoter in H3R117A LoVo cells, while no differences were detected in enrichment of H3K9me3 among the groups (Fig. 6). These results demonstrated that mono-ADP-ribosylation of H3R117 resulted in chromatin microenvironment of TET1 promoter maintained low local poly-ADP-ribosylation, low H3K4me3 level, and high H3K27me3 level. Interestingly, decreased enrichment of PARP1 on *TET1* promoter was discovered in H3R117A LoVo cells (Fig. 6), which seemed contradictory with high enrichment of PAR on *TET1* promoter in H3R117A LoVo cells. We would explain the possible reason for this phenomenon in discussion.

### Mono-ADP-ribosylation of H3R117 had no impact on chromatin accessibility of *TFPI2* but depressed chromatin accessibility of *TET1*

**Fig. 7:**
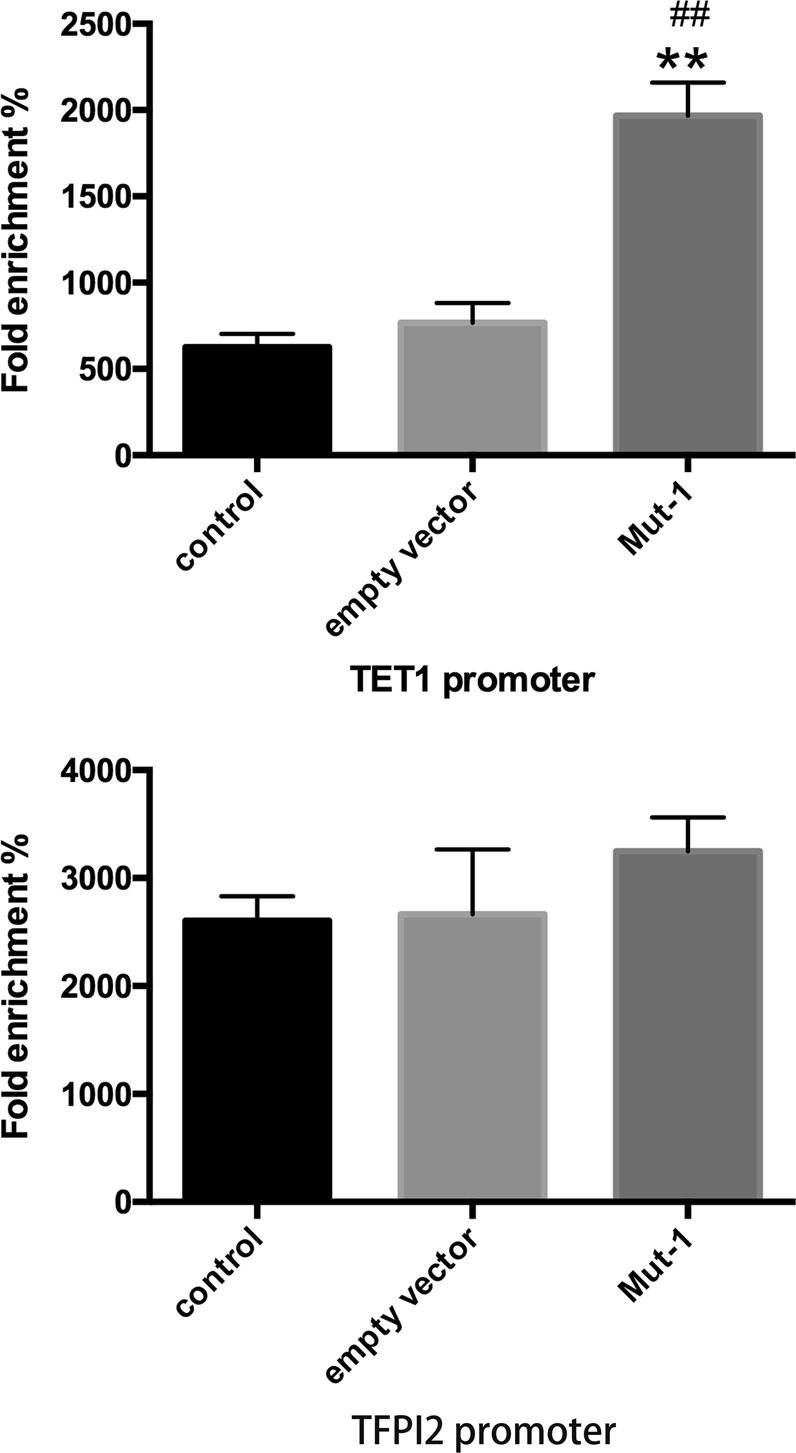
Mono-ADP-ribosylation of H3R117 inhibited chromatin accessibility of *TET1* but not *TFPI2* promoter. Data were presented as the fold enrichment % of amplification efficiency of the Nse-treated DNA sample over that of the No-Nse sample: fold enrichment % = 2^(Ct Nse − Ct No-Nse)^ × 100% (**P < 0.001 vs control, ^##^*P* < 0.001 vs empty vector)

Moreover, to explore whether mono-ADP-ribosylation H3R117 impacted chromatin structure at *TFPI2* and *TET1* promoter, chromatin accessibility assay was applied, and data were presented as the fold enrichment % of amplification efficiency of the Nse-treated DNA sample over that of the No-Nse sample. There was no distinct difference in fold enrichment % at the *TFPI2* promoter among H3R117A LoVo cells, untreated, and empty vector-transfected LoVo cells; however, we detected significantly higher fold enrichment % at the *TET1* promoter in H3R117A LoVo cells than in control and empty vector-transfected LoVo cells (Fig. 7), indicating that mono-ADP-ribosylation of H3R117 contributed to condensation of *TET1* promoter in LoVo cells but not condensation of *TFPI2* promoter.

### H3R117A mutation reducing methylation of *TFPI2* promoter and increasing secretion of TFPI2 via TET1 in LoVo cells

**Fig. 8:**
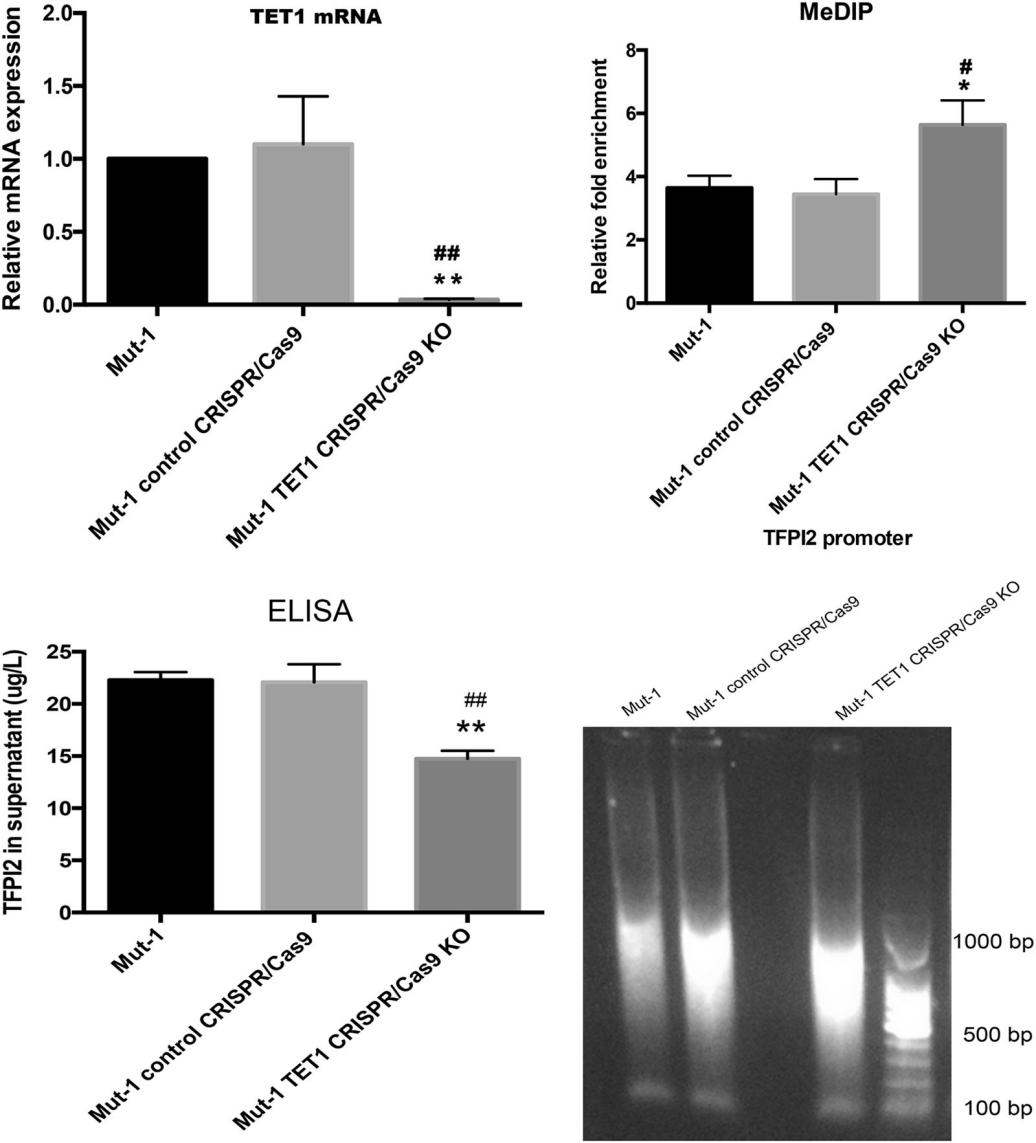
TET1 knockout (KO) of H3R117A LoVo cells by TET1 CRISPR/Cas9 plasmid boosted methylated modification of *TFPI2* promoter and inhibited secretion of TFPI2. Quantitative PCR was applied for confirming TET1 KO after transfection of TET1 CRISPR/Cas9 plasmid, then, DNA was extracted from cultured cells and sheared into length of 200–1000 bp DNA by sonication. Sheared DNA immunoprecipitated with anti-5mC and amplified with primer specific for *TFPI2* promoter. Relative fold enrichment was calculated as the ratio of amplification efficiency of the Methylated DNA Immunoprecipitation (MeDIP) sample over that of non-immune IgG as of the following the formula: relative fold enrichment = 2^(Ct input − Ct sample)^/2^(Ct input − Ct IgG)^. Enzyme-linked immunosorbent assay (ELISA) kit was also used to detected whether secretion of TFPI2 was impacted by TET1 KO in H3R117A LoVo cells, and absorbance was measured by ELISA reader at optical density 450 nm (**P < 0.001 vs control, ^##^*P* < 0.001 vs empty vector, *P < 0.05 vs control, ^#^*P* < 0.05 vs empty vector)

To investigate whether TET1 contributed to attenuation of methylation of *TFPI2* promoter in H3R117A mutant LoVo cells, TET1 CRISPR/Cas9 KO plasmid was used to inhibit expression of TET1 in H3R117A mutant LoVo cells. As indicated in Fig. 8, TET1 CRISPR/Cas9 KO plasmid was confirmed efficiently downregulating TET1 mRNA expression. Moreover, raising methylated modification of *TFPI2* promoter was presented after transfecting TET1 CRISPR/Cas9 KO plasmid into H3R117A mutant LoVo cells (Fig. 8), which demonstrated that H3R117A mutation cut down methylated modification of *TFPI2* promoter depended on increasing of TET1 expression in LoVo cells. Besides, after transfecting TET1 CRISPR/Cas9 KO plasmid into H3R117A mutant LoVo cells the secretion of TFPI2 was detected much lower than H3R117A mutant LoVo cells and control CRISPR/Cas9 group (Fig. 8), suggesting that increasing secretion of TFPI2 in H3R117A mutation LoVo cells due to increasing of TET1 as well.

### Mono-ADP-ribosylation of H3R117 promoted proliferation of LoVo cells and H3R117A mutation depressed proliferation of LoVo cells depended on TET1

**Fig. 9:**
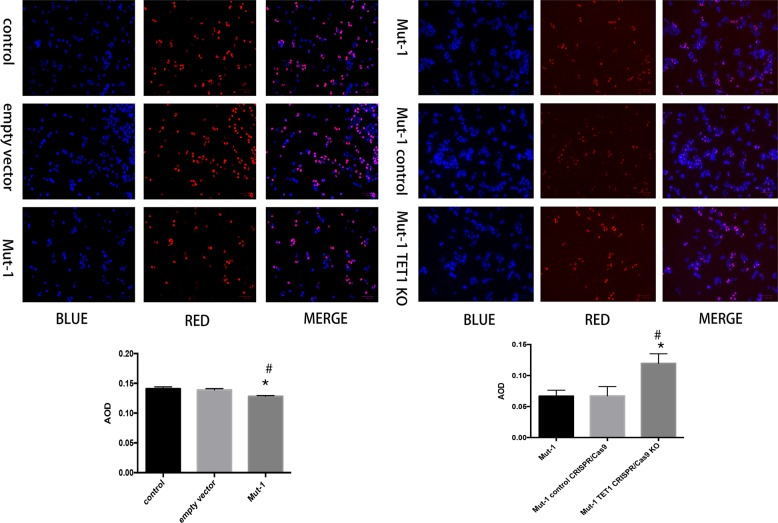
H3R117A mutation depressed proliferation of LoVo cells depending on TET1. Edu assay was applied to detect the consequence of H3R117A mutation on proliferation of LoVo cells, meanwhile, we knockout TET1 in H3R117A LoVo cells to determine the contribution of TET1 to this consequence. The average optical density (AOD) was measured by ImageJ software (*P < 0.05 vs control, ^#^*P* < 0.05 vs empty vector)

Our previous study confirmedlower proliferation of H3R117A mutant LoVo cells by CCK-8, soft agar cloning, and flow cytometry compared with LoVo cells [23]. In this study, we applied Edu assays to evaluate the role of mono-ADP-ribosylation of H3R117 in increasing proliferation of LoVo cells, further investigated whether TET1 contributed to depression of proliferation in H3R117A mutant LoVo cells. Consistent with our previous research, H3R117A mutant LoVo cells showed lower proliferation comparing with control LoVo cells and empty vector group (Fig. 9). Nevertheless, the proliferation of H3R117A mutant LoVo cells was enhancing again after KO TET1 by transfected with TET1 CRISPR/Cas9 KO plasmid (Fig. 9), indicated that mono-ADP-ribosylation of H3R117 indeed increased proliferation of LoVo cells, and TET1 was facilitate to inhibit proliferation of LoVo cells.

### Hypermethylation of *TFPI2* was a ubiquitous trend and early event in CRC, and TET1 was positive to TFPI2 expression in normal and malignant tissue

**Fig. 10:**
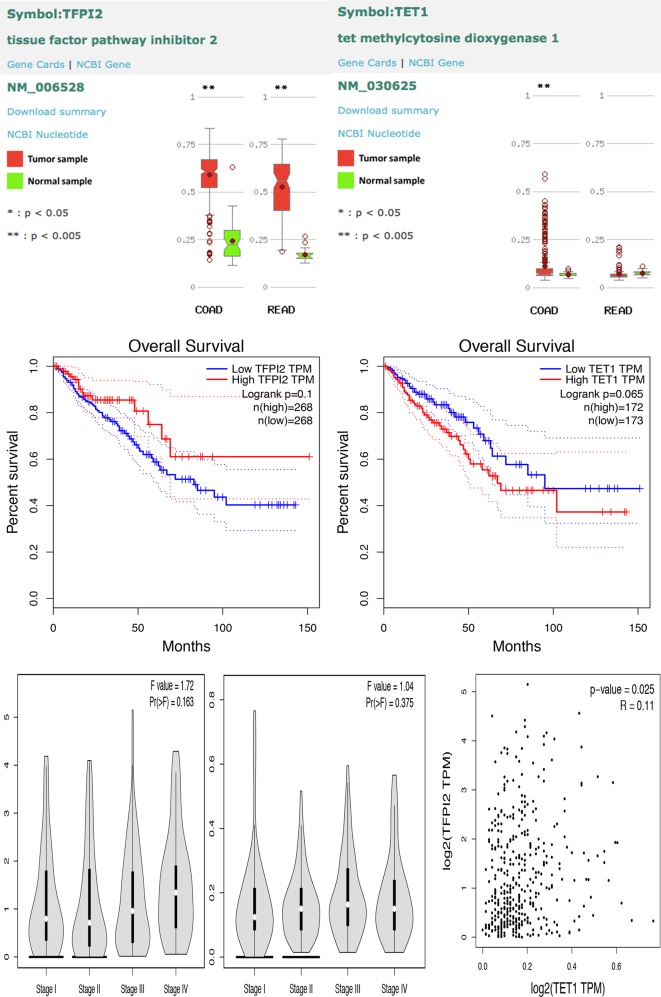
Bioinformatics analyses for abnormal methylation of *TFPI2* and *TET1* in colorectal cancer (CRC), as well as correlation and prognosis influence of transcript level of TFPI2 and TET1 of CRC and normal tissue. Methylated modification of *TFPI2* and *TET1* in normal and CRC was performed using MethHC database, while influence of TFPI2 and TET1 transcript level on overall survival and cancer stage classification of CRC as well as the correlation of these two genes was performed by Gene Expression Profiling Interactive Analysis (the cutoff % of high and low TFPI2/TET1 TPM was 50%)

The bioinformatics results showed that hypermethylation of *TFPI2* was a common phenomenon in CRC comparing with normal tissue according to the The Cancer Genome Atlas (TCGA), and hypermethylation of *TET1* was also detected in colon cancer with respect to normal tissue, but this phenomenon was not observed in rectal cancer (Fig. 10). However, the overall survival was no obvious difference between high TFPI2 or TET1 transcript level and low TFPI2 or TET1 transcript level groups, moreover, different stages of CRC patients appeared no different transcript level of TFPI2 and TET1 (Fig. 10), indicated that hypermethylation of TFPI2 in CRC was an early event. Besides, positive, but not high, correlation of TET1 and TFPI2 expression was detected in normal and malignant tissue according to the TCGA data (Fig. 10), suggesting that TET1 at least partially positively regulated TFPI2 expression.

## Discussion

Recently, nuclear poly-ADP-ribosylation had been noticed as an epigenetic marker, due to its influence on DNA methylation, histone modification, and chromatin remodel, all the core histones could be modified by ADP-ribose, but primarily mono-ADP-ribosylation or short oligomers of ADP-ribose [19]. In our previous research, mono-ADP-ribosylation of H3R117 was identified in human colon carcinoma cell line (LoVo cells) by LC-MS/MS, and this post-translational modification might promote the proliferation of colon carcinoma cells in vitro and in vivo, furthermore, H3R117A LoVo cells were successfully constructed to prevent ADP-ribosylation occur on this amino-acid location [23], however, the function of mono-ADP-ribosylated specific amino acid of histone for epigenetics is still unknown.

In this study, we investigated the contribution of mono-ADP-ribosylation of specific amino acid H3R117 to hypermethylation of TSG *TFPI2*, and discovered mono-ADP-ribosylation of H3R117 facilitated methylation of TSG *TFPI2* and inhibited product of TFPI2 protein in LoVo cells. Besides, we found more TET1 enriched on *TFPI2* promoter in H3R117A LoVo cells rather than control or empty vector-transfected LoVo cells. Owing to TET1 was the dioxygenases catalyze conversion of 5mC to 5hmC [8]; thus, enrichment of TET1 on *TFPI2* promoter generated conversion of 5mC and diminished methylated modification of *TFPI2* promoter in H3R117A LoVo cells. On the contrary, mono-ADP-ribosylated H3R117 protected methylation of *TFPI2* in LoVo cells by depressing TET1 binding on promoter. Although poly-ADP-ribosylation was associated with DNA methylation [26–28], there was no difference of poly-ADP-ribosylation on *TFPI2* promoter between mono-ADP-ribosylated H3R117 LoVo cells and H3R117A LoVo cells, suggesting that mono-ADP-ribosylation of H3R117 did not impact local poly-ADP-ribosylation of *TFPI2* promoter. Thus, we excluded in influence of poly-ADP-ribosylation on DNA methylation of *TFPI2*.

On the other hand, we discovered that histone methylation on *TFPI2* promoter was also impacted by mono-ADP-ribosylated H3R117. Puriti et al. found that H3K9me3-marked genes were protected from methylation changes resulted from TET1 [24], therefore, relative higher level of H3K9me3 of LoVo cells prevented TET1 binding on *TFPI2* promoter DNA, subsequently, maintain hypermethylation of *TFPI2* by protecting 5mC from hydroxymethylation, leading to depression of demethylation of *TFPI2* promoter in mono-ADP-ribosylated H3R117 LoVo cells. Besides, we observed a strong enrichment of H3K27me3 level on *TFPI2* promoter in non-mutated LoVo cells, which was reduced after H3R117A mutation, combining with hypermethylation of *TFPI2* promoter in non-mutated LoVo cells and decreased methylation after H3R117A mutation. These findings were in line with previous reports; promoters that were marked with H3K27me3 are more likely to show increased DNA methylation during differentiation and carcinogenesis than those lacking H3K27me3 [29–31]; thus, hypermethylation of *TFPI2* promoter in mono-ADP-ribosylated H3R117 LoVo cells was also associated with strong enrichment of H2K27me3 at the*TFPI2* promoter. However, because of no difference in poly-ADP-ribosylation, the reason for H3R117A mutation leading to change in chromatin microenvironment on *TFPI2* promoter is still obscure. Further research needs to be done.

Meanwhile, we examined diminished hydroxymethylated modification of *TFPI2* promoter in H3R117A LoVo cells as well. Together with the result of methylation detection on *TFPI2* promoter, indicated that 5hmC was an epigenetic marker independent of 5mC, however, the function of 5hmC seemed obscure so far. Recently, considerable evidences had indicated that the presence of 5hmC at the gene promoter negatively regulates gene expression [32–34]. Therefore, low hydroxymethylated modification of *TFPI2* promoter might another mechanism to reactive *TFPI2* in H3R117A LoVo cells. Interestingly, even *TFPI2* promoter was enriched for TET1 in H3R117A LoVo cells, a decreased hydroxymethylation at these loci was observed in H3R117A LoVo cells in our study. Although all of TET1, TET2, and TET3 catalyzed conversion of 5mC to 5hmC, however, TET2 or TET3 played a major role in 5hmC removal, moreover, 5hmC removal by TET2 was associated with genes enriched for H3K4me3 [24]. Therefore, our data showed that H3R117A mutation in LoVo cells presented reduced 5hmC level with TET1 occupation on *TFPI2* promoter, probably on account of H3K4me3 enrichment inducing 5hmC removal by TET2. Further research needs to be done.

In addition, boosting transcription and expression of TET1 were observed in H3R117A mutant LoVo cells, demonstrating that mono-ADP-ribosylated H3R117 indeed contributed to inhibit TET1 expression in LoVo cells. Besides, abundant enrichment of PAR was measured on *TET1* promoter in H3R117A mutant LoVo cells. In other words, our data demonstrated that mono-ADP-ribosylated H3R117 not only changed local poly-ADP-ribosylation of *TET1* promoter but also the transcription and expression of TET1. Due to multi-level effects of poly-ADP-ribosylation on epigenetic events [26–28], we analyzed chromatin environment on *TET1* promoter to explore whether change of local poly-ADP-ribosylation induced by H3R117A mutation impacted relevant epigenetic events on *TET1* promoter.

First, DNA methylation was a modulator of gene transcription. When located in a gene promoter, DNA methylation typically acts to repress gene transcription. In our research, we found decreasing methylated modification on *TET1* promoter in H3R117A mutant LoVo cells versus control LoVo cells. According to Ciccarone’s report that poly-ADP-ribosylation of local chromatin modulated DNA methylation of *TET1* gene [20], we speculated that attenuated methylation of *TET1* promoter in H3R117A LoVo cells was associated with increased local poly-ADP-ribosylated modification, while mono-ADP-ribosylated H3R117 resulted in abnormal methylation of *TET1* gene by reducing poly-ADP-ribosylated modification on *TET1* promoter.

Second, the nucleosome limited the accessibility of many regulatory factors. In general, the more accessible the DNA fragments the more likely surrounding genes were actively transcribed. Martinez-Zamudio et al. revealed that histone ADP-ribosylation facilitates gene transcription by destabilizing histone–DNA interaction and increasing the site accessibility of nucleosomal DNA to nucleases [22]. Consistently in our study, enrichment of PAR on *TET1* promoter encouraged TET1 transcription by decondensation chromatin of *TET1* promoter in H3R117A LoVo cells, while mono-ADP-ribosylated H3R117 played an opposite role to depress transcription of TET1 in non-mutated LoVo cells. On the other hand, chromatin accessibility of *TFPI2* promoter was not changed, probably due to no significant difference of PAR on *TFPI2* promoter.

In agreement with *TET1* transcriptional state, ChIP assay evidenced a raised enrichment of the active transcription marker H3K4me3 on *TET1* promoter in H3R117A LoVo cells. Therefore, mono-ADP-ribosylated H3R117 mutation changed original status of low H3K4me3 enrichment on *TET1* promoter, and facilitated *TET1* transcription in some degrees. Namely, LoVo cells were not enriched for the active transcription mark H3K4me3 on *TET1* promoter with mono-ADP-ribosylated H3R117. It was proved that poly-ADP-ribosylation of the histone lysine demethylase KDM5B blocked its binding to chromatin, thus restrained demethylation of H3K4me3 [35]. Hence, we inferred mono-ADP-ribosylated H3R117 decreasing local poly-ADP-ribosylation of *TET1* promoter, which allowed activity of KDM5B and diminished H3K4me3 modification on *TET1* promoter. Besides, Yamaguchi et al. recently reported that poly-ADP-ribosylation of EZH2 induced PRC2 complex dissociation and EZH2 downregulation, which in turn reduces EZH2-mediated H3K27 trimethylation [36]. This conclusion explained mono-ADP-ribosylated H3R117 mutation resulted in relative low H3K27me3 level with more poly-ADP-ribosylation on *TET1* promoter, probably due to increasing poly-ADP-ribosylation impacted function of EZH2, which in turn reduced EZH2-mediated H3K27me3. While H3K27me3 was considered as a repressive transcription mark, thus, low H3K27me3 level of TET1 promoter could be one of the reasons for increasing expression of TET1 in H3R117A LoVo cells.

The evidences above indicated that due to limited poly-ADP-ribosylated modification on *TET1* promoter in LoVo cells with mono-ADP-ribosylated H3R117, DNA methylation of *TET1* was increased while chromatin accessibility and H3K4me3 level on *TET1* promoter were inhibited. These epigenetic events could abate transcription and expression of TET1 in LoVo cells with mono-ADP-ribosylated H3R117.

Interestingly, we found dropped enrichment of PARP1 on *TET1* promoter H3R117A LoVo cells, which seemed conflict with increased poly-ADP-ribosylation on this DNA fragments. Nevertheless, PARP1 was not the only PAR polymerase involve in nuclear poly-ADP-ribosylation. PARP2 was also been proved localized in the nucleus and might account for the residual PAR synthesis when PARP1 was deficient [37]. Additionally, Ciccarone et al. showed lower TET1 mRNA level in PARP2-silenced cells rather than PARP1-silenced cells [20]. These evidences suggested that PARP2 played a more important role on transcription of TET1. Thus, we conjectured that it was PARP2 instead of PARP1 mainly responsible for poly-ADP-ribosylation on *TET1* promoter in H3R117A LoVo cells. But verification test needs to be done.

To prove the key role of TET1 on modulating methylation of *TFPI2* promoter, TET1 was knockout (KO) by TET1 CRISPR/Cas9 KO plasmid in H3R117A LoVo cells. Our results showed that even H3R117A mutation reduced methylation of *TFPI2* promoter, after KO TET1 of H3R117A LoVo cells *TFPI2* promoter regained methylated modification, suggesting that TET1 was facilitated of demethylation of *TFPI2* promoter in LoVo cells. Likewise, secretion of TFPI2 was suppressed in TET1 CRISPR/Cas9 KO plasmid-transfected H3R117A LoVo cells, demonstrating that TET1 enhances secretion of TFPI2. Consistent with our previous study [23] we applied Edu assay also to prove mono-ADP-ribosylated H3R117 promoted proliferation of LoVo cells, besides, we observed proliferation of H3R117A LoVo cells was recovered after KO TET1. Since secretion of TFPI2 was also attenuated after TET1 KO, considering TFPI2 was a tumor suppressor of CRC and had been reported inhibited proliferation [38], we inferred H3R117A inhibited proliferation of LoVo cells by regulating TET1-mediated secretion of TFPI2.

Interestingly, we found that hypermethylation of *TFPI2* was prevalent in CRC comparing with normal tissue, but the transcription of TFPI2 seemed no influence on overall survival and staging according to bioinformatics analyses, neither transcription of TET1. These data manifested that hypermethylation of *TFPI2* was early event of tumorigenesis, which consistent with Rasmussen’s point that hypermethylated *TFPI2* was associated with recurrence and early stage of CRC [6], like a switch of turning on tumor. Thus, reversing hypermethylation of *TFPI2* at early stage of cancer, probably an efficient pathway to inhibit tumorigenesis. Bioinformatics analyses was also showed the transcription of these two genes had a positive correlation with each other in CRC and normal tissue, suggesting TET1 at least partially influenced TFPI2 expression in CRC and normal tissue.

In summary, in this study we demonstrated that mono-ADP-ribosylation of H3R117 limited local poly-ADP-ribosylation of *TET1* promoter. This limitation impacted following epigenetic events, increased methylation level, and inhibited chromatin accessibility of *TET1* promoter as well as H3K4me3 level on *TET1* promoter, which reduced transcription and expression of TET1 in LoVo cells. Subsequently, less TET1 were provided to hydroxymethylate 5mC. On the other side, mono-ADP-ribosylation of H3R117 maintained H3K9me3 modification on *TFPI2* promoter, which isolated targets of *TFPI2* promoter from TET1. Thus, mono-ADP-ribosylation of H3R117 exhausted expression of TET1 and H3K9me3-dependent depressed affinity of TET1 to *TFPI2* promoter, impairing demethylation of TET1, coupled with high H3K27me3 level of *TFPI2* promoter to recruit DNA methylation, thus contributing to abnormal methylation of *TFPI2*. Moreover, methylation of *TFPI2* was boosted and secretion of TFPI2 was reduced after TET1 KO in H3R117A LoVo cells, further proved effect of TET1 on *TFPI2* methylation and expression.

However, since mono-ADP-ribosylation of H3R117 is a genome modification rather than one nucleosome, further research on analysis of epigenetic changes of genome especially TSGs and oncogenes resulting from mono-ADP-ribosylation of H3R117 can shed light on the role of mono-ADP-ribosylation of H3R117 in tumorigenesis. Besides, the impact of mono-ADP-ribosylation of H3R117 on the expression and function of TET2 and TET3 was not investigated in this study. Although we have discussed the possible function of TET2 in hydroxymethylation of *TFPI2*, further study needs to be done. Nevertheless, our study first highlighted the epigenetic function of mono-ADP-ribosylation of H3R117 on abnormal methylation of TSG *TFPI2* by impact TET1, since hypermethyaltion of *TFPI2* was an early event in tumorigenesis, selectively target mono-ADP-ribosylation of H3R117 deficiency could be a feasible way to block tumorigenesis of CRC.

## Materials and methods

### Cells

Human colon adenocarcinoma LoVo cells were kindly obtained from Professor Wei-Xue Tang, Chongqing Medical University (Chongqing, China), while point mutation H3R117 LoVo cells and empty vector-transfected LoVo cells were successfully constructed in our previous study [21]. All these types of LoVo cells were cultured in Dulbecco’s modified Eagle’s medium (Hyclone, Logan, UT, USA) with 10% fetal bovine serum (Hyclone) at 5% CO2 and 37 °C.

### Primary antibodies

The following primary antibodies were used in this study: anti-TET1 antibody (GeneTex, GTX627420), anti-H3 antibody (GeneTex, GTX122148), anti-PAR (Trevigen, 4335-MC-100), anti-PARP1 (Cell Signaling Technology, 9532), anti-H3K9me3 (Cell Signaling Technology, 13969), anti-H3K4me3 (Cell Signaling Technology, 9727), and anti-H3K27me3 (Cell Signaling Technology, 9733).

### Primer sequences

Oligonucleotide primers were designed as follows:

TET1 mRNA forward primer TGCCCCACATTGATGAGTATTG,

TET1 mRNA reverse primer AGAGGCGGGTTGGATGATTAC,

Actin mRNA forward primer CACCATTGGCAATGAGCGGTTC,

Actin mRNA reverse primer AGGTCTTTGCGGGTGTCCACGT,

*TFPI2* promoter forward primer CTGATTCATGCACGGGGACT,

*TFPI2* promoter reverse primer CCCGTCTGGACTACAGGAGA,

*TET1* promoter forward primer CAAGTCATGCAGCCCTACCT,

*TET1* promoter reverse primer GGCTCCCAGCACAGTCAA.

### Western blotting

Western blotting was performed on nuclear protein lysates, which was extracted by Nuclear protein lysates Nuclear and Cytoplasmic Protein Extraction Kit (Beyotime, China). The concentration of protein was detected by BCA protein assay (Beyotime, China). Then, proteins were separated by weight by SDS-polyacrylamide gel electrophoresis, and transferred onto polyvinylidene fluoride membrane (Millipore, MA, USA). Following pre-incubation with a blocking solution, membranes were incubated in the primary antibody (mouse monoclonal anti-TET1 antibody) at 4 °C (1:500 dilution) overnight. The membranes were then extensively washed, incubated in secondary antibody (1:2000 dilution, ABclonal, China), and were visualized using ChemiDoc XRS (Bio-Rad, USA). Relative protein levels were obtained using Quantity One^®^ software (Bio-Rad, USA) and were normalized to H3 (1:1000 dilution).

### Isolation of total RNA and reverse transcription

Total RNA was isolated using CellAmp^TM^ Direct RNA Prep Kit for RT-PCR (Takara, China). Then, complementary DNA was synthesized with the directions of the PrimeScript^TM^RT reagent Kit (Perfect Real Time) (Takara, China).

### Quantitative PCR

The quantitative real-time PCR followed the directions of the SYBR® Premix Ex Taq™ II (Takara, China), using oligonucleotide primers for human TET1. Actin was used as reference gene for quantification. Quantification was carried out using the geometrical average of 2^−△△Ct^ for TET1 relative to Actin.

### MeDIP-qPCR and hMeDIP-qPCR

5mC immunoprecipitation was carried out using the EpiQuik MeDIP Kit (Epigentek, NY, USA) according to the manufacturer’s manual. This kit included a ChIP-grade 5mC antibody and a negative control normal mouse IgG. Briefly, DNA in the cells was extracted and sheared into 200–1000 bp fragments. An aliquot of each sample was set aside as input control, while the remaining portion was subjected to immunoprecipitate with 5mC antibody or normal mouse IgG. DNA was released from the antibody-DNA complex by Proteinase K and purified through the specifically designed Fast-Spin Column. Eluted DNA was amplified with primer specific for *TFPI2* promoter or *TET1* promoter. Relative fold enrichment presented enrichment of 5mC on *TFPI2* promoter: relative fold enrichment = 2^(input Ct − sample Ct)^/2^(input Ct – IgG Ct)^.

For hMeDIP-qPCR, 5hmC immunoprecipitation was carried out using the EpiQuik hMeDIP Kit (Epigentek), according to the manufacturer’s recommendations. This kit included a positive control DNA fragment, a negative control non-immune IgG, and control primers that can be used with the positive control to demonstrate the enrichment efficacy for hydroxymethylated DNA with the kit reagents and protocol. The positive control DNA containing 5hmC can be immunoprecipitated by a 5hmC antibody but not by a non-immune IgG. Briefly, DNA in the cells was extracted and sheared into 200–1000 bp fragments as described in MeDIP Kit. Sample DNA and positive control DNA were added into the microwell immobilized with 5hmC or IgG antibody, respectively. DNA was released from the antibody-DNA complex by add Proteinase K. Finally, eluted DNA was amplified with primer specific for *TFPI2* promoter or *TET1* promoter. An input DNA control was generally not needed as the included positive and negative controls could be used for estimating the same objective more accurately. Fold enrichment % was calculated by simply using a ratio of amplification efficiency of the ChIP sample over that of non-immune IgG: fold enrichment % = 2^(IgG Ct – Sample Ct)^ × 100%. The sensitivity of the methods was estimated by analyzing the reference DNA fragment containing 5hmC provided by the kit.

### Chromatin accessibility

Chromatin assembly was carried out using the EpiQuik chromatin accessibility assay kit (Epigentek, NY, USA). In this assay, first, cell lysis and chromatin extraction were performed. Then, chromatin was digested with or without a nuclease (Nse) mix, and purified by spin column. Eluted DNA was amplified using qPCR and gene-specific primer for *TFPI2* promoter or *TET1* promoter. Control primers were provided to determine the successful digestion of the chromatin. The fold enrichment was calculated by the ratio of amplification efficiency of the Nse-treated DNA sample over that of the control sample not treated with nuclease (No-Nse): fold enrichment % = 2^(Nse Ct − No-Nse Ct)^ × 100%. Changes in chromatin structure were identified by the degree of Ct shift between digested and undigested samples.

### Chromatin immunoprecipitation

ChIP assays were carried out following the protocol from the SimpleChIP Enzymatic Chromatin IP Kit (Cell Signaling, 9002). Briefly, cells were treated with 1% formaldehyde for 10 min at room temperature to crosslink proteins to DNA. The chromatin was harvested and digested to a length of approximately 150–900 bp fragments using micrococcal nuclease, then, sonicate lysate with several pulses to break nuclear membrane. An aliquot of each sample was set aside as input control, while the remaining portion was subjected to immunoprecipitation with anti-TET1 (5 μl/IP), anti-PAR (5 μl/IP), anti-PARP1 (2 μl/IP), anti-H3K9me3 (10 μl/IP), anti-H3K4me3 (5 μl/IP), or anti-H3K27me3 (10 μl/IP) overnight at 4 °C, with IgG (5 μl/IP) as negative control. The complex of co-precipitation was captured by ChIP-Grade Protein G Agarose Beads, and chromatin was eluted from antibody/Protein G Beads and reversed crosslinks. Subsequently, DNA was purified and eluted for qPCR. DNA was amplified by PCR using primer pairs designed to amplify TFPI2 promoter or TET1 promoter.

### Generation of TET1 CRISPR/Cas9 KO cells

To generate TET1 KO cells, TET1 CRISPR/Cas9 KO Plasmid (h) (Santa Cruz) was transfected into the corresponding H3R117A mutant LoVo cells according to the manufacturer’s instructions, and control CRISPR/Cas9 Plasmid (Santa Cruz)-transfected cells were considered as empty control. Then, 48–72 h after transfection, qPCR analysis was performed to confirm TET1 KO of H3R117A mutant LoVo cells.

### Enzyme-linked immunosorbent assay

Quantitative analysis of TFPI2 concentration was respectively conducted by using TFPI2 ELISA kit (Jiangsu Mbbiology Biological Technology Co., Ltd, China) following the manufacturer’s instructions. The absorbance was measured by an ELISA reader (Multiskan FC microplate photometer, Thermo Fisher Scientific) at OD 450 nm.

### Immunofluorescence

Cells were fixed in 4% paraformaldehyde at room temperature for 30 min and then permeabilized using 0.5% Triton X-100. Then, cells were washed with cold phosphate-buffered saline to remove free Triton X-100. Subsequently, samples were incubated with primary antibody TET1 at 4 °C overnight. Cells were washed and incubated with Cy3-conjugated secondary antibody (Proteintech, SA00009-1) for 1 h and counterstained with 4′,6-diamidino-2-phenylindole (for nuclear staining). Immunofluorescence was assessed under ZOETM Fluorescent Cell Imager (Bio-Rad) using ImageJ software (version 1.8.0; National Institutes of Health, USA). The assay was repeated in triplicate.

### Edu assay

The EdU detection kit (Beyotime, China) was used to evaluate cell proliferation. According to the manufacturer’s protocol, cells were treated with 20 μM EdU for 2 h at 37 °C and fixed with 4% paraformaldehyde at room temperature for 15 min. Following washing with wash buffer three times, cells were treated with 0.5% Triton X-100 for 15 min and stained with Click reaction cocktail for 30 min at room temperature. Following washing with wash buffer, 1× Hoechst 33342 dye was used to incubate cells at room temperature for 10 min. Images were captured using ZOETM Fluorescent Cell Imager (Bio-Rad). Following the merging of the images, the average OD of cells was calculated using ImageJ software (version 1.8.0; National Institutes of Health, USA). The assay was repeated in triplicate.

### Bioinformatics analyses

Methylated modification of *TFPI2* and *TET1* in normal and CRC was performed using MethHC [39], a database of DNA methylation and gene expression in human cancer, which is freely accessible at http://methhc.mbc.nctu.edu.tw/

The influence of TFPI2 and TET1 transcript level on prognosis of CRC patients was performed by Gene Expression Profiling Interactive Analysis (GEPIA), which was developed by Zefang Tang, Chenwei Li, and Boxi Kang of Zhang Lab, Peking University [40]. GEPIA is a newly developed interactive web server for analyzing the RNA sequencing expression data of 9736 tumors and 8587 normal samples from the TCGA and the GTEx projects, using a standard processing pipeline. GEPIA is available at http://gepia.cancer-pku.cn/.

### Statistical analysis

Experiments were replicated at least thrice. Values were presented as mean ± standard deviation (*x* ± *s*). Statistical analyses were performed using the GraphPad Prism 6.0 (GraphPad Software, Inc., CA, USA). One-way analysis of variance was adopted, with a significance level defined as *P* < 0.05.
